# Cost-effectiveness analysis of COVID-19 tests in the unified health system

**DOI:** 10.1186/s12962-023-00469-1

**Published:** 2023-09-13

**Authors:** Vinicius Queiroz Miranda Cedro, Stéfany de Lima Gomes, Ana Clara Correa Duarte Simões, Tatiana do Valle Lovato Sverzut, Keila Cristina Xavier Bertti, Marcelo Tadeu Tristão, Yuri Wanderley Cavalcanti, João Victor Frazão Câmara, Antonio Carlos Pereira

**Affiliations:** 1 Department of Community Dentistry, Piracicaba Dental School, Universidade Estadual de Campinas - UNICAMP, Piracicaba, SP Brazil; 2 Department of Clinical and Social Dentistry, Federal University of Paraíba, João Pessoa, Paraíba, Brazil; 3 Clinic of Operative Dentistry, Periodontology and Preventive Dentistry, Saarland University Hospital, Homburg/Saar, Germany

**Keywords:** Cost-effectiveness evaluation, COVID-19, Unified Health System

## Abstract

**Background:**

To evaluate the cost-effectiveness ratio and economic impact of the Rapid Antigen Test (TR-Ag) to replace RT-PCR for the detection of the new Coronavirus in the Unified Health System (SUS).

**Methods:**

This is a cost-effectiveness analysis. Clinical protocols were used for the diagnosis of COVID-19 at the São José Municipal Hospital, located in the city of Itaberá-SP. The Incremental Cost-Effectiveness Ratio (ICER) was divided into two scenarios. In the first, the accuracy reported by the test manufacturers was included, and in the second, the cost resulting from a systematic review. Both were compared with the performance of the RT-PCR test. The increase in diagnoses was chosen as a health outcome and absenteeism was used as a criterion for assessing the economic impact.

**Results:**

The analysis resulted in incremental cost-effectiveness ratios of R$ 42,136.67 and R$ 68,329.73 for every thousand tests, according to the accuracy of the manufacturers’ TR-Ag tests and what is reported in the literature in relation to RT-PCR, respectively. The average value found for the RT-PCR test (R$ 202.87) represents an increase of 165.32% in cost in relation to the value found for the TR-Ag. 4,305 tests were performed between April 2020 and December 2021 at the referral hospital. Also, maintaining the use of RT-PCR as the first choice for diagnosing COVID-19 and regulating absenteeism in the economically active population could have an impact of up to R$ 1,022,779.68 on municipal management.

**Conclusion:**

It is concluded that the TR-Ag are configured as a cost-effective alternative for the SUS in the detection of the new Coronavirus. The strategy becomes economically favorable for the expansion of testing, combating the COVID-19 pandemic and reducing the impact on the local economy. However, studies are needed to validate the accuracy of the tests so that economic evaluations on the subject are more assertive.

## Introduction

SARS-CoV-2 is a new betacoronavirus belonging to the viral family of coronaviridae, first identified in an outbreak of pneumonia cases in Wuhan City, Hubei Province, China, in December 2019 [1]. The International Committee on Taxonomy of Virus (International Committee on Taxonomy of Viruses) named the new Coronavirus, which means in English Severe Acute Respiratory Syndrome Coronavirus 21. The disease caused by SARS-CoV2, called COVID-19, was declared a pandemic in March 2020 by the World Health Organization (WHO), presenting the world with a new epidemiological scenario [2].

According to WHO data, as of March 15, 2022, 458,479,635 cases of COVID-19 were confirmed worldwide, with 6,047,653 deaths recorded [3]. In Brazil, the first case was confirmed in the city of São Paulo, in February 2020, and since then, 29,380,063 confirmed cases have been reported, with 655,249 related deaths as of March 14, 2022 [4]. The transmission of COVID-19 occurs when a person infected with the virus shares droplets with people nearby by coughing, sneezing, or maintaining direct contact by shaking hands, followed by mucosal contact [5].

Some strategies are being used to face COVID-19, including the performance of the diagnostic test as a form of monitoring and surveillance [6]. This technology allows countries to develop strategic plans for coping with the pandemic according to their epidemiological situation, imposing well-defined objectives and using the resources available in their territory [7]. Currently, the laboratory tests available for the identification of the SARS-CoV-2 virus use techniques such as the Reverse Transcription Polymerase Chain Reaction (RT-PCR), rapid antigen detection tests (TR-Ag) or those that detect antibodies., with indications according to the course of the infection [6].

RT-PCR is considered the gold standard test for detecting SARS-CoV-2. The technique was developed at the Charité Institute in Berlin in January 2020 and identifies viral RNA in samples collected by swab from the nasal cavity and oropharynx. On the other hand, antigen tests look for proteins on the viral surface, and their samples are collected by means of a nasopharyngeal smear, anterior nostrils or saliva. The sample is exposed to paper strips containing artificial antibodies designed to bind to Coronavirus antigens. The antigens bind to the strips and provide a visual readout. This process takes less than 30 min and can provide point-of-care results and does not require expensive equipment or extensive training [8].

Thus, the quick tests present easier execution, with less time and cost to obtain the result. The disease can be detected soon after the first days of symptoms appear, not requiring a complex laboratory structure. However, these tests offer lower sensitivity and specificity when compared to RT-PCR and, sometimes, they must be repeated [9]. On the other hand, RT-PCR has better accuracy, even in the first days of infection, even before the onset of symptoms. As disadvantages, these tests have a higher cost, require a more complex laboratory structure and longer time to process the results [9, 10].

In this highly complex scenario, the public manager responsible for offering health care is questioned about which intervention is the most effective to be implemented in the territory of its responsibility, in view of the limitation of resources faced in the Unified Health System (SUS) [11]. In view of this, Economic Health Assessments (EHA) are presented as a support tool for the provision of health services and can be classified as partial or complete [12]. In the complete health evaluations, a comparison is made between the costs and the health outcome of at least two investigated alternatives. There are types of complete analysis: cost-minimization, cost-effectiveness, cost-utility and cost-benefit. These analyzes provide managers with information that is not described in the cost of the disease and are used for more assertive interventions, contributing to a rational choice in the application of resources [13].

Therefore, the notorious importance of detecting patients infected by SARS-CoV-2 for the control of the pandemic, there is a limited availability in the literature of economic analysis that guide the use of technology with the best cost-effectiveness ratio for this economic scenario [14]. Given the high availability of technologies and different brands available, decision-making by the public administration becomes complex [15]. Thus, this study is an important guiding evidence for decision makers, whose objective was to evaluate the economic impact of the use of TR-Ag tests, instead of RT-PCR, through a cost-effectiveness analysis.

## Methods

### Planning the economic analysis in health and ethical aspects

The study project was submitted to the Research Ethics Committee of the Piracicaba Dental School (Universidade Estadual de Campinas (CEP – FOP/UNICAMP) CAAE n. 42567021.2.0000.5418. Informed consent was obtained from all patients and/or their legal guardian(s).

This is an economic analysis, carried out through the use of the cost-effectiveness analysis strategy, with the objective of evaluating different tests for the detection of the new Coronavirus used during the COVID-19 pandemic. To carry out the cost effectiveness analysis, the Methodological Guidelines for Economic Assessment and Technical-Scientific Opinions of the Ministry of Health [13, 16] were used. The study was written according to the international guideline Consolidated Health Economic Evaluation Reporting Standards Statement (CHEERS) [17]. All methods were carried out in accordance with relevant guidelines and regulations (Declaration of Helsinki).

### Population

This economic analysis considered SUS users as the target population for the detection of the virus that causes COVID-19.

The analysis was performed with secondary data, based on information provided by the Hospital Municipal São José (HMSJ), in consultation made by the researchers. In the treatment of information, all users who sought the system to perform the diagnostic test in the SUS were included. There was no exclusion of groups in the sample of the target population. All data identifying patients were excluded.

### Municipality profile

This study was developed from the context of the service provided by HMSJ, an institution belonging to the Unified Health System (SUS), located in the city of Itaberá, state of São Paulo, Brazil, during the COVID-19 pandemic. SUS was created in 1988 with the objective of guaranteeing the health of all Brazilians. In order to promote quality healthcare, the Federal Constitution established that the system should be ensured through social and economic policies that promote the reduction of diseases and other health issues, making it the only public healthcare system that serves over 200 million people. Financed by taxes collected from citizens, the system is constantly evolving to ensure universality, equality, and comprehensiveness for its citizens [11]. Due to the health, social and economic context generated by the COVID-19 pandemic, caused by the novel Coronavirus (SARS-CoV-2), the resilience of SUS has been tested with a significant increase in demands across its various levels of care [18]. Furthermore, the Ministry of Health assessed the accuracy of several diagnostic tests registered for COVID-19 in 2020. At that time, no studies validating the accuracy of the tests were found, and information on the sensitivity and specificity of the exams was solely described by the manufacturers [15].

Based on the geometric mean of the three dimensions of the Human Development Index (income, longevity and education), the Municipality’s Human Development Index is calculated. Itaberá’s IDHM is 0.69, which is considered average. The average infant mortality rate in the city is 21.28 per 1,000 live births. In 2020, the average monthly salary was 2.1 times the minimum wage. Considering households with monthly income of up to half the minimum wage per person, 41.1% of the population were in these conditions, which placed it in position 32 out of 645 among the cities in the state [19].

### Comparators

The Incremental Cost-Effectiveness Ratio (ICER) was divided into two scenarios for comparison. In the first, the TR-Ag accuracy reported by the manufacturers was included, and in the second, the value resulting from a systematic review [15, 18]. Both were compared with the performance of the RT-PCR test. The test marks were reported by the HMSJ. For both tests, samples are collected from a nasopharyngeal swab, the reference method for collecting diagnoses [8, 20].

### Study perspective and scenarios

The perspective of this study is that of the HMSJ manager, responsible for controlling and combating the COVID-19 pandemic. The positive and negative predictive values were not provided by the manufacturer, thus, the information was not made available for accurate description and comparison [15]. Concerning the comparison between groups, the total population was compared with the scenario of a sample of 24.7% of patients using private health plans, as well as the formal occupancy rate being 19.6%, according to estimates by the Brazilian government in 2021 [19].

### Time horizon, currency and discount rate

The non-longitudinal analysis time reference was developed for the economic scenario of the COVID-19 pandemic. All costs were estimated in Reais (R$), in average values for 2021 and not exceeding the 12-month analysis did not suffer discount rates or inflationary corrections.

### Resource estimation

The costs were collected, using a micro-costing approach, in three stages: the identification of costs, the measurement of the quantity and the respective value [13]. The identification of the necessary inputs for the execution of the tests was provided by the HMSJ, through a direct request and approved by the ethics committee, which were listed and statistically analyzed using the Microsoft Excel program.

The protocol informed by the HMSJ recommended the use of: (i) a non-sterile surgical procedure glove by a professional for each test performed; (ii) one semi-facial mask, type N95/PFF2, per professional on each shift; (iii) a disposable protective cap, per professional on each shift; (iv) a disposable and waterproof lab coat, per professional on each shift; (v) a disposable and waterproof sneaker, per professional on each shift; (vi) one protective eyewear, per professional, for use during one semester; (vii) one face shield, per professional, for use during one semester.

Some assumptions were adopted to quantify the resources used. Hygiene items were recommended according to the quantity described in the study by Cavalcanti et al., due to the compatibility in the assistance provided for the care of patients with COVID-19: (viii) 50mL of 70% alcohol for cleaning surfaces per shift; (ix) 20mL of liquid soap for cleaning hands and arms per shift; (x) 32 sheets of paper towels for drying hands and arms per shift; (xi) 1 sanitary mat for shoes, for use by professionals and patients, per semester [21].

Human resources costs were reported by the HMSJ, which defines a 12-hour work shift. The team consists of a doctor, nurse and nursing technician. The values ​​were adjusted for a journey of 7 working days per week, 365 per year. The 12-hour shift was divided into 1 h to prepare the testing room, 1 h break for food and rest, and 10 h of useful work.

The amount of 60 tests is recommended for each team on duty in the period of 12 h, in order to stipulate maximum work efficiency. The time to perform the tests includes the reception, identification, evaluation and medical advice, collection, processing of TR-Ag or processing of the RT-PCR sample in the laboratory.

### Price research

The monetary values ​​of the TR-Ag and RT-PCR tests were obtained through the COVID-19 Procurement Panel of the Federal Government Procurement Portal [22]. The average costs of inputs through the Price Panel of the Ministry of Economy [23]. Cats on medical shifts, nurses and nursing technicians were informed by the municipality’s transparency portal [24]. The average value of the Brazilian salary was obtained in an Executive Summary of the Ministry of Labor and Social Security [25].

### Health outcome

The increase in diagnosis of COVID-19. As a comparator, the sensitivity, specificity and accuracy of TR-Ag were obtained from the manufacturers’ manuals and the literature. They consider the RT-PCR as an accuracy reference and, therefore, for this study, the samples were considered positive if the results with the reference method were positive (reagents) and considered negative (non-reactive) if the results with the reference were negative [8, 26, 27]. The literature available in the databases (PubMed, Scielo, Google Scholar, Medline) was used to carry out the research, without limitation of date or language. The terms “Accuracy” and “COVID-19” and “Test” were used. The data from the manufacturers of the TR-Ag tests were obtained from the product manuals, available in the public consultation system of the National Health Surveillance Agency [15].

### Methods for calculating incremental cost-effectiveness ratio and resource allocative efficiency

The assessment of the Incremental Cost-Effectiveness Ratio (ICER) was defined by the difference between the cost of TR-Ag and RT-PCR over the difference in the accuracy of the same tests [12]. The final value was multiplied by one thousand for the purpose of relative population comparison. The following formula demonstrates the adapted ICER calculation for this study:

1 $$ICER = \left( {\frac{{Cos{t_{TR - Ag}} - Cos{t_{RT - PCR}}}}{{{\text{Accurac}}{{\text{y}}_{TR - Ag}} - {\text{Accurac}}{{\text{y}}_{RT - PCR}}}}} \right) \times 1000$$

The evaluation of the economic-financial impact was carried out based on data from patients tested by the HMSJ, through the Municipal Health Department of Itaberá. Data relevant to the day of the symptom, test day and result date were used.

For comparison purposes, it was recommended that patients tested with RT-PCR received an initial medical certificate of 5 days to await the test result in social isolation, while those tested with TR-Ag were released immediately after a negative result. The value of the average Brazilian salary was used to determine the financial impact on the municipality [25].

### Sensitivity analysis

There was no willingness-to-pay (WTP) threshold for diagnostic tests in Brazil, however, for this analysis, the researchers set the WTP at R$ 100.00, a value under advanced discussion in technology assessment forums in Brazil, and the use of Net Monetary Benefit (NMB) was used to assess the benefit of the technologies. For the sensitivity, specificity and accuracy of the tests, a 95% confidence interval was estimated. For the sensitivity analysis, the variation of costs (± 10%) and accuracy rates (± 5%) of the RT-PCR and TR-Ag tests was considered, in order to present an optimistic, probable and pessimistic expectation about the allocative efficiency of the resources. In this scenario, it is important to note that the accuracy cannot be higher than 100%. Therefore, we consider the accuracy of the TR-Ag test as 100%. It is important to note that a 5% variation is high, but this variation is realistic and consistent with the reality of testing in the historical context of the pandemic. We explore how these variations may affect the conclusions regarding the choice of test, using Microsoft Excel, so that a relative analysis can be reflected.

## Results

The total cost per patient for diagnostic testing of COVID-19 through RT-PCR was R$ 202.87 (± 10% 182.58–223.16) (Table 1). The same diagnosis performed using TR-Ag cost R$ 76.46 (± 10% 68.82–84.11) per patient according to the protocol investigated in the study (Table 1). The main input costs for patients were identified as R$ 54.26 (± 10% 48.83–59.69), human resources R$ 157.22 (± 10% 141.50–172.94) (Table 1). Over a year, the total cost for carrying out diagnostic tests with TR-Ag is R$ 1,674,474.46 (± 10% 1,339,579.57–2,009,369.35) and R$ 4,442,853.46 (± 10% 3,554,282.77–5,331,424.15) with RT-PCR, representing a difference of R$ 2,768,379.00 (± 10% 2,214,703.20–3,322,054.80) in the total cost of the tests (Table 1).

**Table 1:** Description of the inputs, quantities and costs necessary to carry out the diagnostic tests for COVID-19 performed at the HMSJ.

Materials and inputs	Use	Quantity per shift	Number of uses	Average price	Amount	Unit price	Total cost per shift	Total cost per year
**Pairs of gloves**	PPE for team	360	1	R$ 48.04	100 un	R$ 0.48	R$ 172.94	R$ 63.124.56
**n95 mask**	PPE for team	3	1	R$ 5.98	1 un	R$ 5.98	R$ 17.94	R$ 6.548.10
**Disposable Hat**	PPE for team	3	1	R$ 22.13	100 un	R$ 0.22	R$ 0.66	R$ 242.32
**Disposable Coat**	PPE for team	3	1	R$ 48.49	10 un	R$ 4.85	R$ 14.55	R$ 5.309.47
**Foot protector**	PPE for team	6	1	R$ 38.40	100 un	R$ 0.38	R$ 2.30	R$ 840.96
**Protective glasses**	PPE for team	3	65.700 (per year)	R$ 4.56	1 un.	R$ 4.56	R$ 0.01	R$ 3.65
**Face shield**	PPE for team	3	65.700 (per year)	R$ 37.73	1 un.	R$ 37.73	R$ 0.01	R$ 3.65
**70% alcohol**	Surface cleaning	50 mL	8	R$ 6.69	1000 mL	R$ 0.01	R$ 0.33	R$ 122.09
**Liquid soap**	Degermation	20 mL	16	R$ 33.43	1000 mL	R$ 0.03	R$ 0.67	R$ 244.04
**Paper towel**	Drying of hands and arms	32	1	R$ 10.93	1000 un.	R$ 0.01	R$ 0.35	R$ 127.66
**Sanitary mat**	Shoe cleaning	1	87.600 (per year)	R$ 66.27	60 × 40 cm	R$ 0.01	R$ 0.01	R$ 3.65
**Nursing Technician**	On duty	1	12 horas	R$ 122.82	12 horas	R$ 10.24	R$ 122.82	R$ 44.829.30
**Nurse**	On duty	1	12 horas	R$ 323.80	12 horas	R$ 26.98	R$ 323.80	R$ 118.187.00
**Doctor**	On duty	1	12 horas	R$ 1.440.00	12 horas	R$ 120.00	R$ 1.440.00	R$ 525.600.00
			**Partial Cost per Patient**:			R$ 34.94	R$ 2.096.40	R$ 765.186.46
				**Total RT-PCR Cost**:		R$ 202.87	R$ 12.172.20	R$ 4.442.853.46
				**Total Cost TR-Ag**:		R$ 76.46	R$ 4.587.60	R$ 1.674.474.46
			**Test Cost Difference**:			R$ 126.41	R$ 7.584.60	R$ 2.768.379.00

Table adapted by the authors.^21^

The sensitivity, specificity and accuracy of the rapid tests used in the study were reported by the manufacturers of the tests used in the HMSJ and the reference values ​​in the literature through a systematic review [20]. The mean accuracy presented in the manual of the TR-Ag manufacturers was 98.15%, while the average found in the literature was 97%, for the same indicator when compared to the reference test (Table 2) [27]. Also, the average found in the literature for the RT test -PCR, considered the gold standard test, was 92.3% (Table 2) [20].

**Table 2:** Sensitivity, specificity and accuracy of diagnostic tests for COVID-19.

Test	Sensitivity (%) 95% CI	Specificity (%) 95% CI	Accuracy (%)
**Wondfo** **(TR-Ag)** ^**15**^	96.18% (96,43%-98,49%)	99.72% (98.45-99.95%)	97.67%
**VivaDiag** **(TR-Ag)** ^**15**^	95.04% (89,60%-97,71%)	100% (99.12-100%)	97.87%
**ECO Test** **(TR-Ag)** ^**15**^	96.49% (CI not informed)	99.03% (CI not informed)	98.91%
			98.15%*
**TR-Ag (Literature)** ^**27**^	69% (68-70%)	99% (99%-99%)	97%
**RT-PCR (Literature)** ^**20**^	81.4% (70-90%)	100% (96-100%)	92.3%

Table produced by the authors. Average Accuracy Provided by Manufacturers of TR-Ag*

Through the comparative evaluation of the testing strategies, in which the reference value (RT-PCR) was compared with the cost and effectiveness found in the TR-Ag investigated in the study, it was possible to verify two Incremental Cost-Effectiveness Ratios. The ICER found, for every thousand exams, through comparison with the literature data was R$ 42,136.67 (± 10% 37,923.00–46,350.34) and the relation with the data reported by the manufacturers was R$ 68,329.73 (± 10% 61,496.76–75,162.70) (Table 3). These values ​​represent an additional expense for performing the RT-PCR. Furthermore, the incremental accuracy for the molecular test is 3% and 1.85%, respectively.

**Table 3:** Test accuracy and incremental cost-effectiveness ratio

Test	Cost (R$) ± 10%	Δ Cost (R$) ± 10%	Accuracy (%) ± 5%	Δ Accuracy (%) ± 5%	RCEI (R$ x 1000) ± 10%	NMB** ±5%		
**TR-Ag (Literature)** ^**27**^	R$ 76.46 (68.82–84.11)	-	97% ( 92 − 100%)	-	-	20.54 (19.51–21.57		
**RT-PCR**	R$ 202.87 (182.58–223.16)	R$ -126.41 (113.77–139.05)	100% (Reference)*	-3% (2.85% − 3,15%)	R$ 42.136.67 ( 37,923.00 - 46,350.34)	-102.87 (-97.73 - -107.01)		
**TR-Ag (Manufacturers)** ^**15**^	R$ 76.46 (68.82–84.11)	-	98.15% (93.24 − 100%)	-	-	21.69 (20.61–22.77)		
**RT-PCR**	R$ 202.87 (182.58–223.16)	R$ -126.41 (113.77–139.05)	100% (Reference)*	-1.85% (1.76% − 1,94%)	R$ 68.329.73 (61,496.76 - 75,162.70)	-102.87 (-97.73 - -107.01)		

**Table produced by the authors. The values ranged from** ± **10% for costs and** ± 5% for accuracy* The accuracy of the rapid antigen tests was compared with the RT-PCR reference standard. Samples were considered reactive if the results with the reference method were reactive and considered non-reactive if the results with the reference method were non-reactive. ** Net Monetary Benefit.

The average salary of Brazilians in 2021 was BRL 1,921.19 [25]. For a journey of 220 h per month and 8 h per day, the cost is estimated at R$ 69.86 per hour worked. For the RT-PCR test, 5 days of social isolation are required until the diagnostic conclusion. This period represents an estimated productivity reduction of R$ 349.31 over the 5 days and corresponds to R$ 349,307.27 (± 10% 279,445.82–384,237.99) accumulated for every thousand tests performed.

4,305 diagnostic tests were performed between April 2020 and December 2021 at the HMSJ, of which 823 were considered reactive, 3470 were non-reactive and 5 were invalid. The hospital did not report the distribution by test type. 568 patients were over 60 years of age, 2928 were between 18 and 59 years old. The mean age of the patients tested was 36 years. The value of the economic evaluation, defined by the reduction of labor productivity in the population between 18 and 59 years old, can represent an impact of up to R$ 1,022,779.68 (± 10% 920,501.71–1,125,057.65) for the economy in the municipality of Itaberá.

## Discussion

The increase in assertive diagnoses of COVID-19 proved to be an important outcome for the study, given that the new Coronavirus is the most responsible for respiratory tract infections worldwide in the current health scenario [28]. In the present study, the incremental cost-effectiveness ratios were calculated at R$ 42,136.67 (± 10% 37,923.00–46,350.34) and R$ 68,329.73 (± 10% 61,496.76–75,162.70) for every thousand tests, when compared to the accuracy value provided by the literature and by the manufacturers, respectively. The TR-Ag showed lower values ​​for sensitivity (69–96%), and specificity close to the reference test (99.03–100%). The accuracy found ranged from 97 to 98.91%, demonstrating a satisfactory performance for the initial diagnosis of the disease. The WHO recommends a minimum performance of 80% sensitivity and 97% specificity [29].

The incremental cost to perform each RT-PCR is R$126.41 (+ 165.32%). However, it was not possible to find the ICER between the accuracy described in the literature for the RT-PCR, as the test was more expensive and lower values than the TR-Ag. In this case, a false perception that the reference test would have lower efficacy would apply. For a proper assessment, the accuracy values of RT-PCR and TR-Ag values should be compared with a third assessment method, such as CT scan associated with clinical examination. Therefore, this strategy is treated as an absolutely dominated relationship, in which it must be eliminated from the study according to methodological guidelines [13].

In relation to indirect costs, significant attention is drawn. If the RT-PCR test were used for all 2,928 patients who were of average working age, between 18 and 59 years, at least 14,640 days of absenteeism would be expected, which may represent an additional cost mark close to R$ 1,022,779.68 (± 10% 920,501.71–1,125,057.65) for the local economy. It should be noted that the study does not consider the impact on the family group or circle of people who had contact with the suspected patient. These should also be tested and instructed to carry out social distancing, which would result in a jump in the number of people away from work activities [10].

With regard to sociodemographic data, the municipality of Itaberá had an estimated population of 17,405 people (2021) and the Gross Domestic Product per capita estimated at R$ 33,740.36 (2019), according to the Brazilian Institute of Geography and Statistics (BIGS). The proportion of people with formal occupation is 19.6%. According to this projection, at least 3,411 workers between the ages of 18 and 59 could benefit from rapid tests if the entire population is assisted by SUS. However, if the scenario of 75.3% of patients is users fully dependent on SUS, that is, who do not have private health insurance, the workers would be reduced to 2.568, in which TR-Ag could represent a saving of R$ 106,563.63 (24.7%) [19].

The detection of infectious agents with security level 2 (NB2), such as the new Coronavirus, requires specific conditions of laboratory structure and human resources. Professionals must be trained, places must have safety restrictions and the use of Personal Protective Equipment must be mandatory [10]. Thus, when compared to RT-PCR, rapid antigen tests (TR-Ag) have advantages, as they do not require a complex laboratory structure and extensive training, are cheaper, samples can be processed at the point of care and the result is displayed in up to 30 min. The patient receives immediate medical advice and can be directly released for their daily activities. Furthermore, these tests are especially used in the evaluation of return to work [10, 27, 30, 31].

Thus, knowing the stage of the disease is extremely important for the choice of technology and the application of the diagnostic test. An early diagnosis test for infection with the new coronavirus acts on the quality of care provided by the health service and is the first step towards interrupting the chain of transmission of the virus. It is important to point out that laboratory diagnostic tests have several challenges, which start from their availability, means of collection, processing and clinical interpretation, and that faulty diagnostic results are harmful to health care [8].

According to the Pan American Health Organization (PAHO), timely and accurate testing is the key strategy for preventing and controlling the spread of SARS-CoV-214. Diagnosing COVID-19 is critical for confirmed cases to have an appropriate outcome, especially as it relates to patient management and isolation. A false negative result can increase the transmission rate, as well as morbidity and mortality [10]. For this, a high accuracy of the exams should be expected. Until the conclusion of this article, none of the brands analyzed had an independent assessment of accuracy published on the regulatory agency’s website [10, 32]. In addition, in the methodology of this economic analysis, assumptions applied in the provision of the HMSJ service and in the study by Cavalcanti et al. [21]. These specifications may present limitations in the generalization of research results, as well as the wide availability of diagnostic test brands [15].

It should be noted that the reference test is also prone to failure, and its accuracy can be impacted, causing an effect on the sensitivity and specificity value chain of other types of diagnostic tests. Several studies have found discrepant values ​​in the performance of the RT-PCR test, which are caused by errors in the collection procedure and sample transport, inadequate time to obtain the test, virus mutations and the host’s immune response [8, 33–36]. In some cases it is necessary to repeat the exam 3 times to properly detect the virus [37]. However, antigen tests also have disadvantages such as variable sensitivity and specificity, generally lower than nucleic acid amplification testing (NAAT), so lower sensitivity means that the negative predictive value is lower than for NAAT, especially in sites with high prevalence of SARS-CoV-2. Confirmatory NAAT testing for positive RDTs is recommended in all low-prevalence settings and for negative RDTs in high-prevalence settings. Also, negative Ag-RDT results cannot be used to remove a contact from quarantine.

Regarding the limitations of the study, this manuscript presents some methodological, structural and parametric uncertainties. The perspective of the study dealt with the pandemic scenario, in which input prices varied at significant levels in Brazil. It was not possible to establish discount rates and inflationary corrections, given the unusual behavior of the time and the short analytical horizon. However, the sensitivity analysis provided a clearer understanding of how variations in cost and accuracy may influence their decision regarding the choice between RT-PCR and TR-Ag tests. Based on the variation in costs, the RT-PCR test remains more expensive than TR-Ag. Therefore, the preference may remain for the TR-Ag test due to the lower cost, even when the analysis is done simultaneously with the variation in accuracy. In this way, other health managers can realize the approximate cost impact on ICER, in which it will be possible to evaluate the implementation of these technologies, according to their budgetary availability, diagnostic effectiveness and local economic impact.

The methods of cost valuation and diagnostic guidelines of the protocols were constantly updated in the face of scientific developments. Since the study was conceived, knowledge of the virus, the disease, prevention and treatment methods have evolved, but it is necessary to understand and deepen the effectiveness of the treatment that was proposed at the time. The parametric values and assumptions assigned can still be replicated, however for the Brazilian reality, in which small municipalities, through the SUS, were responsible for close to all care, it can be described as a parametric uncertainty.

In the present study, the accuracy values ​​were reported by the manufacturers and compared with the literature to eliminate bias in the research. Diagnosing COVID-19 is a complex challenge [6]. In Brazil, the Ministry of Health recommends the choice of RT-PCR and TR-Ag tests for symptomatic patients in the acute phase and that they be collected until the 8th day of symptoms. However, our study demonstrates that TR-Ag can be especially used in the initial phase of the disease for screening and diagnosis, especially in environments where there is a limitation in the laboratory processing capacity [14, 27, 36]. In addition, some studies have shown false results-negatives in significant amount in the first days of infection (0 to 7 days) for RT-PCR [8, 20].

The results of the present research allow managers to create strategies in order to early correct the spread of the virus and promote interventions that improve care during the course of the disease at the lowest cost. The study also highlights the importance of the economic consequences that the choice of health technology has. The use of rapid antigen tests can significantly reduce costs related to public resources applied to health, without sacrificing quality in the accuracy of the tests because compared to traditional technology, TR-Ag increases the use benefit by 120%. This finding aims to improve the diagnostic actions of patients, promote the reformulation of public policies and conduct the practice in health units.

## Conclusions

Rapid antigen tests have an economically attractive relationship in relation to RT-PCR for the detection of the new Coronavirus. The strategy becomes economically favorable for the expansion of testing and the fight against the COVID-19 pandemic, reducing the impact of the disease on the local economy, due to the reduction of indirect costs related to absence from work. However, studies are needed to validate the accuracy of the tests so that economic evaluations on the subject are more assertive.

## Data Availability

All data generated or analysed during this study are included in this published article [and its supplementary information files].
